# Adolescents’ Perspectives on the Drivers of Obesity Using a Group Model Building Approach: A South African Perspective

**DOI:** 10.3390/ijerph19042160

**Published:** 2022-02-14

**Authors:** Gaironeesa Hendricks, Natalie Savona, Anaely Aguiar, Olufunke Alaba, Sharmilah Booley, Sonia Malczyk, Emmanuel Nwosu, Cecile Knai, Harry Rutter, Knut-Inge Klepp, Janetta Harbron

**Affiliations:** 1 Research Centre for Health through Physical Activity, Lifestyle & Sport, Division of Physio-Logical Sciences, Department of Human Biology, Faculty of Health Sciences, University of Cape Town, Cape Town 7925, South Africa; sharmilah.booley@uct.ac.za (S.B.); soniamalczyk@gmail.com (S.M.); emmanuel.nwosu@uct.ac.za (E.N.); janetta.harbron@uct.ac.za (J.H.); 2 Faculty of Public Health and Policy, London School of Hygiene & Tropical Medicine, London WC1E 7HT, UK; natalie.savona@lshtm.ac.uk (N.S.); cecile.knai@lshtm.ac.uk (C.K.); 3 System Dynamics Group, Department of Geography, University of Bergen, N-5020 Bergen, Norway; anaely.aguiar@uib.no; 4 Health Economics Division, School of Public Health and Family Medicine, University of Cape Town, Cape Town 7925, South Africa; olufunke.alaba@uct.ac.za; 5 Department of Social and Policy Sciences, University of Bath, Bath BA2 7PJ, UK; hr526@bath.ac.uk; 6 Division of Mental and Physical Health, Norwegian Institute of Public Health, N-0316 Oslo, Norway; knut-inge.klepp@fhi.no

**Keywords:** obesity, group model building, adolescents, qualitative, system mapping

## Abstract

Overweight and obesity increase the risk of a range of poor physiological and psychosocial health outcomes. Previous work with well-defined cohorts has explored the determinants of obesity and employed various methods and measures; however, less is known on the broader societal drivers, beyond individual-level influences, using a systems framework with adolescents. The aim of this study was to explore the drivers of obesity from adolescents’ perspectives using a systems approach through group model building in four South African schools. Group model building was used to generate 4 causal loop diagrams with 62 adolescents aged 16–18 years. These maps were merged into one final map, and the main themes were identified: (i) physical activity and social media use; (ii) physical activity, health-related morbidity, and socio-economic status; (iii) accessibility of unhealthy food and energy intake/body weight; (iv) psychological distress, body weight, and weight-related bullying; and (v) parental involvement and unhealthy food intake. Our study identified meaningful policy-relevant insights into the drivers of adolescent obesity, as described by the young people themselves in a South African context. This approach, both the process of construction and the final visualization, provides a basis for taking a novel approach to prevention and intervention recommendations for adolescent obesity.

## 1. Introduction

Overweight and obesity have become a growing concern to researchers, health professionals, and policymakers given their associated health and psychological implications. Identified as the fifth leading risk for global death [1], 2 billion individuals worldwide are overweight or obesity [2], and of this, 62% of the world’s people living with obesity reside in low- and middle-income countries (LMICs) [3]. In South Africa, prevalence rates have shown that between the ages 15 and 19 years, 26.8% of females and 8.6% of males are overweight or obese [4]. Overweight and obesity are associated with adverse health outcomes, with a progressive rise in the prevalence of other chronic diseases, including diabetes, cardiovascular disease, and some forms of cancer [5]. These outcomes may persist throughout adolescence and into adulthood [6]. The diminished health status that often persists throughout adolescence has also been associated with the adoption of unhealthy dietary and physical activity behaviors [6].

A complex myriad of risk factors has been associated with overweight and obesity. Physiological risk factors include genetics, microbiome, brain–gut axis, and basic homeostatic and metabolic alterations [7]. Behavioral risk factors include extensive exposure to electronic screen devices, high consumption of sugar-sweetened beverages, fast-food consumption, and lack of physical activity [8]. Other factors include psychological distress and exposure to stressors, and adverse family or school environments [9]. Increased global trade liberalization, economic growth, and rapid urbanization have contributed to dramatic changes in diets and behavior patterns [9,10,11]. There has been a predominant focus on individual factors; however, much remains unknown, particularly about the systematic factors that impact adolescent overweight and obesity [12].

Previous studies have explored factors associated with obesity among adolescents and have employed various methods [13]; however, one study took a systems approach, using group model building (GMB) [14]. GMB provides a framework to gain new insights into complex systems, such as adolescent obesity, and helps analyze the central drivers of the problem [15]. This is in contrast to traditional approaches, which tend to focus on individual-level factors rather than environmental drivers of obesity [16]. One defining component of GMB is the visual models called causal loop diagrams (CLDs), which provide a ‘map’ of the complexity of a problem comprising multiple interrelated drivers, causal relationships, and polarity (positive or negative direction) [17]. Understanding the effects of complex interactive yet interdependent factors within a connected system is crucial to exploring and identifying the characteristics that are uniquely associated with overweight and obesity in adolescents.

Some such work has, however, taken place in high-income countries (HICs) [14], where only a relatively small percentage of the world’s population lives. Complex interactive risk factor profiles in LMICs may differ from those of developed countries. For example, high levels of malnutrition, ongoing conflict and violence, inequitable distribution of health services, education inequality for girls, and inadequate commitment to adolescents’ participation around issues affecting their lives are more prevalent in LMICs [18]. The high burden and developmental profile of adolescent obesity in LMICs further reinforces the need for a multi-level systems approach that fully engages with and responds to the complexity of the problem. For the purpose of this study, system mapping sessions were implemented using the GMB technique to explore adolescents’ perceptions of the determinants of obesity in four South African schools.

## 2. Materials and Methods 

### 2.1. Design and Setting

This study forms part of a project called Confronting Obesity: Co-creating policy with youth (CO-CREATE), a multi-setting multi-strategy project that aims to reduce the prevalence of obesity among adolescents through policy actions to promote healthier food and physical activity environments. This paper reports on a qualitative research study, including generating CLDs using group model building and comprehensive notetaking, to depict factors perceived by participants as affecting the diet choices and physical activity of adolescents and hence obesity.

The target population was adolescents aged 16–18 years attending 4 public schools in the Western Cape province of South Africa. Participants (N = 62) were recruited from schools from different socio-economic (SES) backgrounds in diverse geographic areas. It is recommended that on average, a GMB session should consist of 15 participants [17]. Participants in the sessions included school #1 = 7; school #2 = 15; school #3 = 18; and school #4 = 22. 

The schools were selected using purposive cluster sampling by SES and geographic area. The Western Cape province has eight education districts (EDs), of which four EDs include schools in the city of Cape Town while the other four EDs include schools outside the capital. Two schools were selected from EDs in Cape Town and two schools were from EDs outside Cape Town. The quantile system that classifies SES of all public schools in SA was used to select two high-SES and two low-SES schools. The schools are categorized into one of five quantiles, with quintile one (Q1) schools generally located in the poorest communities and quintile five (Q5) schools in the wealthiest communities. Q1 to Q3 are no-fees schools while Q4 and Q5 charge school fees. From the four EDs in the Cape Town metropole, we selected one school from schools classified as either Q1, Q2, or Q3 (representing the capital low-SES school) and one school from schools classified as either Q4 or Q5 (representing the capital high-SES school). Similarly, one non-capital low-SES school and one non-capital high-SES school were recruited from the non-capital EDs. Only mixed-gender schools were included to obtain a representative view from both genders. It was also considered that the four schools have learners that represent the variety of cultural and racial groups in the Western Cape province. 

The four schools identified were contacted, informed about the study, and invited to participate. If a school’s headmaster or governing body agreed and provided school consent, adolescents in grade 10 to 12 were informed about the study and asked to volunteer for participation. Adolescents were eligible for the study if they were 16–18 years old at the time of the GMB session, registered in a school, and competent to give assent or consent to participate in the study. Those not fulfilling the age and other requirements were excluded from the study.

### 2.2. Ethics

Ethical considerations, including consent, confidentiality, anonymity, and voluntary participation, were discussed with the adolescents. Information sheets and consent forms were sent home to the parents/caregivers of eligible adolescents that indicated their interest in taking part in the study. Informed consent was obtained from the parents/legal guardians of the adolescents, and assent from the adolescents. The study was approved by the Human Research Ethics Committee (HREC) of the Faculty of Health Sciences of the University of Cape Town (HREC REF: 257/2019). Permission to conduct the study in public schools was obtained from the Western Cape Education Department (WCED). 

### 2.3. Data Collection 

First, 2 sessions of 90 min each were scheduled and conducted at each school to construct the CLDs for this study, and detailed notes were taken to document the discussions. Sessions were held in English, Afrikaans, IsiXhosa, or a combination of the relevant languages. All notes were translated into English. 

This study followed a systematic method using GMB to derive CLDs qualitatively representing the determinants of adolescent obesity. GMB is a well-recognized method for depicting the drivers of obesity, and the complexities it entails, to help guide the development of policy responses [17]. This method is a structured collaborative process designed to guide participants through various stages to generate a causal loop diagram, which depicts the factors they believe contribute to adolescent obesity. A CLD using a GMB approach demonstrates not only the factors in the system but also allows for an in-depth investigation of the causal relationships and connections between the variables. Within CLDs, feedback loops (FBLs) depict complex interactions in a system, and the effect these interactions may have on the system. FBLs represent influential points at which interventions may improve the system’s performance by presenting linkages that may either reinforce or break connections within the system [15]. This system includes a network of FBLs to explain why certain core elements in the system behave as they do.

The process of GMB used for this study [17] necessitates multiple roles to generate and integrate the factors into a digital CLD. During both sessions, participants were guided using tried and tested methods, and session scripts were followed to ensure that the sessions were conducted consistently between the four schools. The facilitators received training from experienced GMB leaders from Deakin University on how to facilitate GMB sessions resulting in informative CLDs and FBLs. For the purpose of this study, at least three facilitators were present at each session. 

Session #1 at each school began with a short slideshow introducing the CO-CREATE project, and its value, by reviewing adolescent overweight and obesity prevalence and discussing the various health implications. This slideshow also introduced the purpose of the sessions, describing various terms, such as ‘policy’, ‘guideline’, and ‘systems focus’. Participants were led through a series of activities as part of session #1, including: individual and group brainstorming on factors that affect overweight/obesity; a ‘graph over time’ exercise to demonstrate how some factors have either increased, decreased, or stayed the same over time i.e., their dynamism; small group sessions for participants to share and prioritize the various factors that they brainstormed; and whole group discussions of the participants’ prioritized factors. Finally, the facilitators used live online software to generate a map showing the factors and their interaction. For the construction of this map, the participants indicated which factors should be connected and whether the relationship between factors is in a positive or negative direction. A software called STICKE (Systems Thinking in Community Knowledge Exchange;(Global Obesity Centre and the Institute for Intelligent Systems Research and Innovation, Deakin University in Melbourne, Australia) was used to digitally generate the CLDs during the GMB sessions with the participants. 

During session #2, the adolescents reviewed a printed version of the map created in the first session and suggested changes following small group discussions. Session #2 also allowed the opportunity for the facilitators to clarify the details and definitions of some factors (e.g., the difference between “junk food”, “take-aways”, “fast foods”, and “oily foods”, according to the participants), and to gain more insight into the meaning of some factors (“stress” relating to pressures from multiple sources, such as academic, financial, relationship, violence, xenophobia, and safety). Participants were then divided into small groups and asked to select a section of the map and brainstorm an “action idea” to help overcome the chain of causation of the problem. Small groups then presented and shared their action ideas to the rest of the group for discussion. 

The maps for each school, and the final merged map of all four schools, followed a system dynamics convention to explain causal relationships [17]. A positive polarity (represented as the ‘solid line’) indicated a positive relationship between the two variables (i.e., as cause increases, the effect increases and as cause decreases, the effect decreases), and a negative polarity (represented as the “dotted line”) indicated an inverse relationship between the two variables (i.e., as cause increases, effect decreases and as cause decreases, effect increases).

Notetaking played a crucial role in the process during the session (and after the session). During the sessions, researchers provide detailed and descriptive field notes of real-time observations. After both sessions were complete in each school, the research team re-engaged with CLDs, comparing the diagrams with their notes, to ensure that the CLD reflected the discussions that took place within the schools. Furthermore, these observational field notes were maintained in a notebook. 

### 2.4. Map Merging

The research team was responsible for generating the maps in STICKE, and for continuous notetaking to document the discussion, which resulted in a final map for each school. The four CLDs (Appendix B, Appendix C, Appendix D and Appendix E) produced by the young people from each school were merged into one ‘master map’. This map merging process included a practical process involving the South African CO-CREATE team (G.H., J.H., O.A. and E.N.) and a member from the United Kingdom CO-CREATE team (N.S.) and Norway CO-CREATE team (A.A.). Several steps were implemented to obtain the final master map according to a systematic process by Savona et al. [14]. Firstly, each CLD was edited to remove variables that had connections going only in or only out. The second step was to select from the four maps the one with the most variables remaining as the ‘base map’. Each variable on the other three maps was examined in relation to the base map and judged to be either: discarded as a duplicate; added to the base; or not fitting anywhere. Some variables were discarded entirely if they did not fit or made sense and other variables were placed to the side for later deliberation. The research team ensured that the variables were correctly represented and checked if any had been unnecessarily discarded. The final map clearly represented the causal links and structures suggested by the adolescents and was checked by all team members for validity to ensure links between FBLs were correctly represented (Appendix A). The nature of GMB is such that the focus is on generating FBLs that depict the factors that the young people believe contribute to obesity. 

Within the final merged map, the research team identified the FBLs that illustrate important drivers of obesity and are potentially important for intervention. Five FBLs that demonstrate key themes in the final map and offer opportunities for policy intervention. 

## 3. Results 

**Figure 1:**
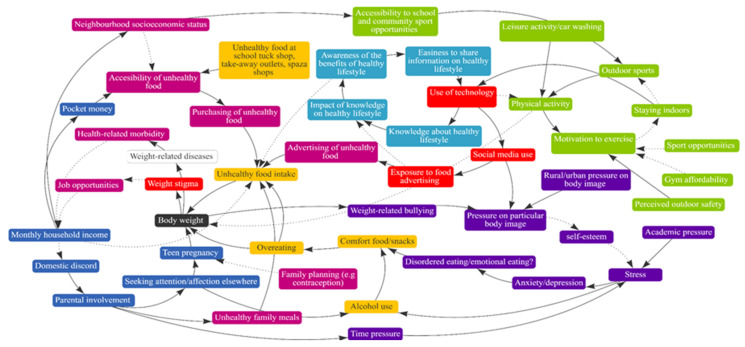
Combined map representing the perceptions of young people of the drivers of obesity in four South African schools.

Each school created one CLD and the four maps were amalgamated into one merged master map. The resulting merged map in Figure 1 illustrates a summary of the 62 adolescents’ views of the drivers of obesity. The variables on the CLD were broadly color-coded into themes. In Figure 1, the green blocks illustrate factors related to physical activity while the red and orange blocks are related to online activities and food/drink, respectively. The light blue blocks are related to knowledge/information while the dark blue is related to home life. The fuchsia color, on the other hand, indicates the economic/commercial influence while the purple blocks are related to mental health, and the black block indicates body weight. 

The five key FBLs that were identified in the final CLD include: (i) physical activity and social media use; (ii) physical activity, health-related morbidity, and socio-economic status; (iii) accessibility of unhealthy food and energy intake/body weight; (iv) psychological distress, body weight, and weight-related bullying; and (v) parental involvement and unhealthy food intake.

**Figure 2:**
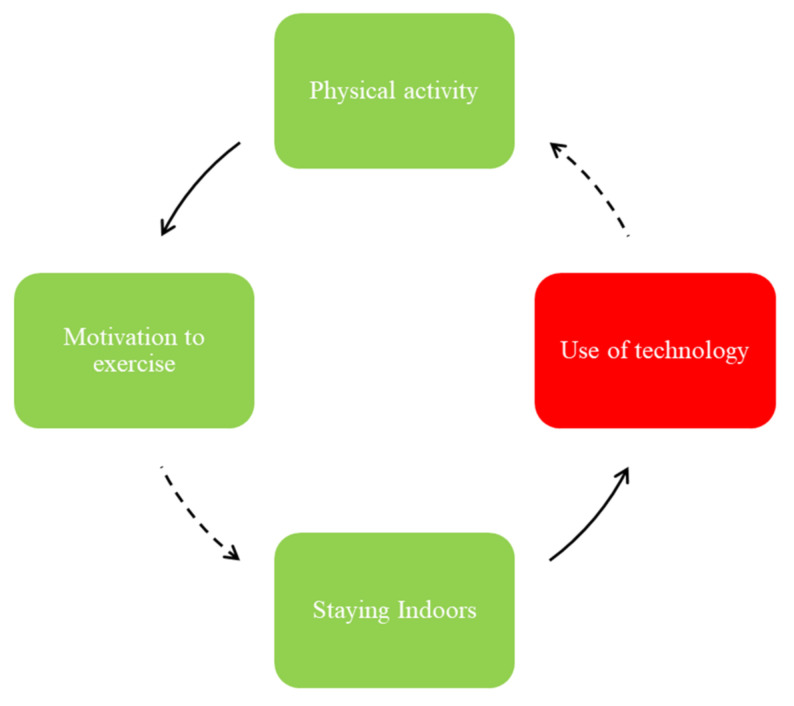
Physical activity and social media use.

One area of focus, as shown in the FBL illustrated in Figure 2, was the role and impact of physical activity in adolescents’ motivation to exercise, staying indoors, and use of technology. 

**Figure 3:**
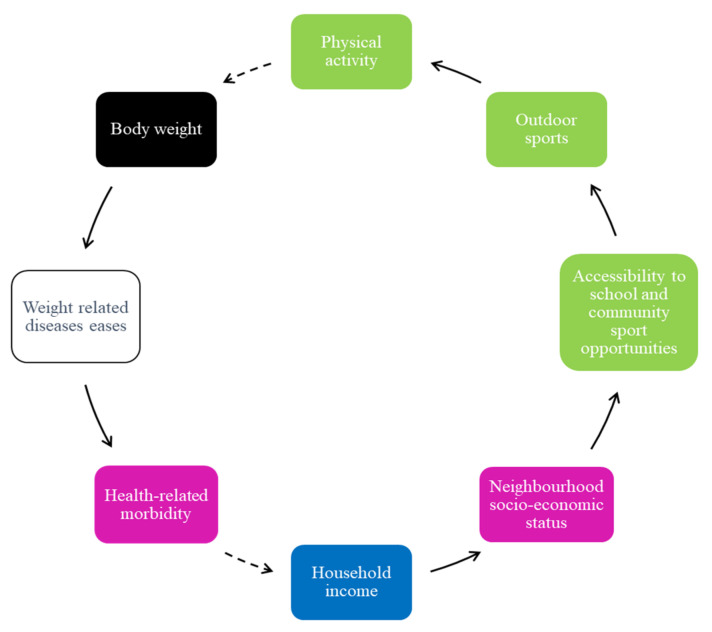
Physical activity, health-related morbidity, and socio-economic status.

While the adolescents reported that engaging in physical activity may enhance their motivation to exercise, this motivation to exercise can be hampered by the aspects of community safety, sports opportunities, and affordability of gyms (Figure 1). While community safety is not demonstrated in Figure 2 and Figure 3, they mentioned feeling unsafe resulting in minimal outdoor activities (e.g., walking, running) and increased exposure to social media and consequently unhealthy food advertisements (see Figure 1). 

In addition, most adolescents described that exercise programs are minimal, especially in low-income neighborhoods, and those that are available are situated far from their place of residence. Adolescents in the schools in low-income neighborhoods also mentioned that the sports opportunities in their schools are often limited to only one type of sport for everyone and that there are no regular practice times or matches and that schools do not employ coaches to train learners (Figure 3). They reported that the lack of accessibility to exercise programs and sports impacts their involvement in outdoor sports. Thus, they reported feeling uninterested in physical activity and preferred staying indoors.

The low level of physical activity contributes to higher body weight, risk of weight-related diseases, and health-related morbidities (Figure 3). Increased health-related morbidity is perceived to influence changes in household income.

**Figure 4:**
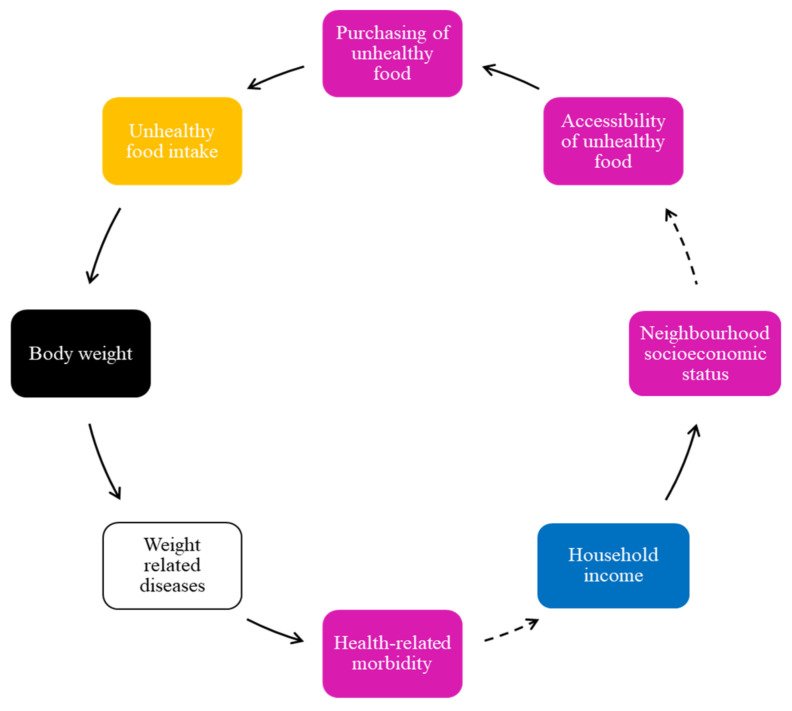
Accessibility of unhealthy food and energy intake/body weight.

Adolescents reported that factors such as unhealthy food, energy intake, and weight gain are influenced by the easy accessibility to unhealthy foods; hence, it is another FBL (Figure 4).

In Figure 4, easy accessibility of unhealthy food contributes to increased purchase and consumption of unhealthy food, and a higher body weight among adolescents. According to the adolescents, advertising of unhealthy foods, including attractive specials of fast-food outlets, which they felt increased their exposure to cheap unhealthy food, placed them at greater risk of not only unhealthy food intake but also weight-related diseases, and additional health-related morbidity. Some reported that those from lower-income households have challenges regarding health-related morbidities, and thus continue to have easy access to unhealthy food in their communities, driving unhealthy food consumption. 

As indicated in Figure 1, easy accessibility to informal convenience shops, such as *tuck shops, spaza shops, or take-out outlets* (a spaza shop, also known as a tuck shop or street foods/vendor, is an informal convenience shop business in South Africa, usually run from a home), that mostly sell unhealthy foods or drinks at affordable prices for the large portion size received is particularly discouraging and makes it easy to purchase unhealthy food. Even though easy access to convenience shops is not illustrated in Figure 3, several adolescents expressed that this accessibility influenced their purchase of unhealthy food and unhealthy food intake. It was mentioned that these shops are located just outside school fences and close to their homes, thus they make purchases during school break times and after school.

**Figure 5:**
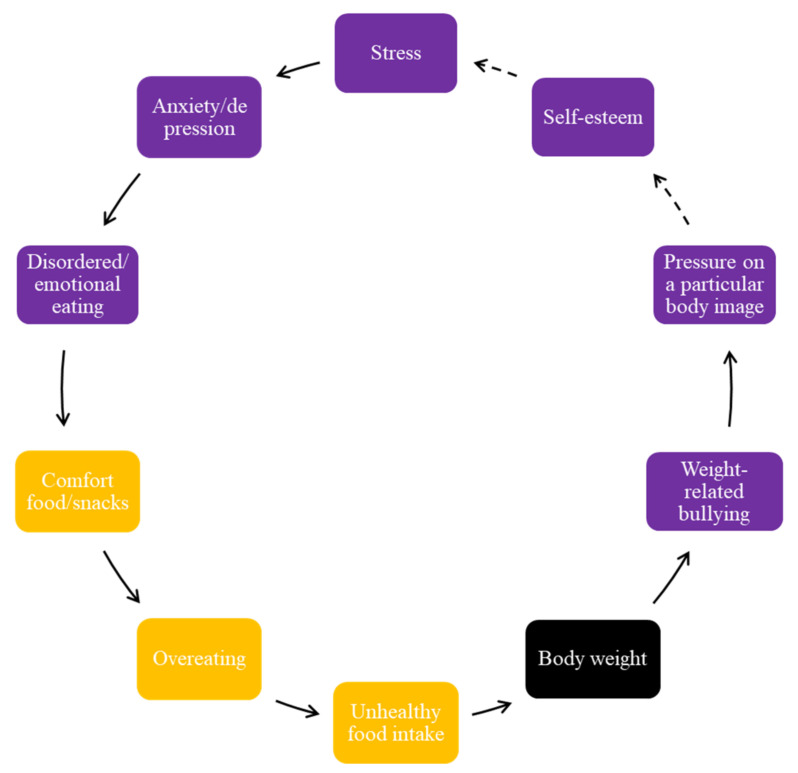
Psychological distress, body weight, and weight-related bullying.

The regular consumption of unhealthy food by adolescents brought on negative feelings about their body weight and negative perceptions of their body image and shape; hence, this is another FBL (Figure 5).

Figure 5 encompasses the concept of psychological distress, and the impact it has on body weight and weight-related bullying (Figure 5). They reported, for example, that specific stressors contribute to weight-related behaviors and emotional eating. All groups mentioned various factors that cause stress in adolescents, including academic work, home environment, and peer influences. Stress triggered mental health conditions, such as depression or anxiety, and subsequent disordered or emotional eating. They reported that these negative emotions influenced their food choices and food intake, cited as either compulsive, addictive, or binge eating, and contributes to a cycle of increased energy intake and body weight. 

Some adolescents mentioned that a greater body weight is stigmatized in their communities and pressure is felt regarding the expectation of a particular body image and shape. This pressure contributes to a cycle of excessive eating and associated mental health issues, and as a result, can lead to weight gain and weight-related bullying. One interesting aspect that emerged is the different standards and expectations of a particular body image within a culture depending on the area of residence. For example, a larger body shape is expected when they return to their extended family over holiday periods, in comparison to an expected thinner/slimmer body shape in the city region where they are in school. Feeling pressure and expectations to have a particular body image and shape was seen as contributing to poor self-esteem, and these expectations, and other mental health challenges, made them vulnerable to additional stress.

**Figure 6:**
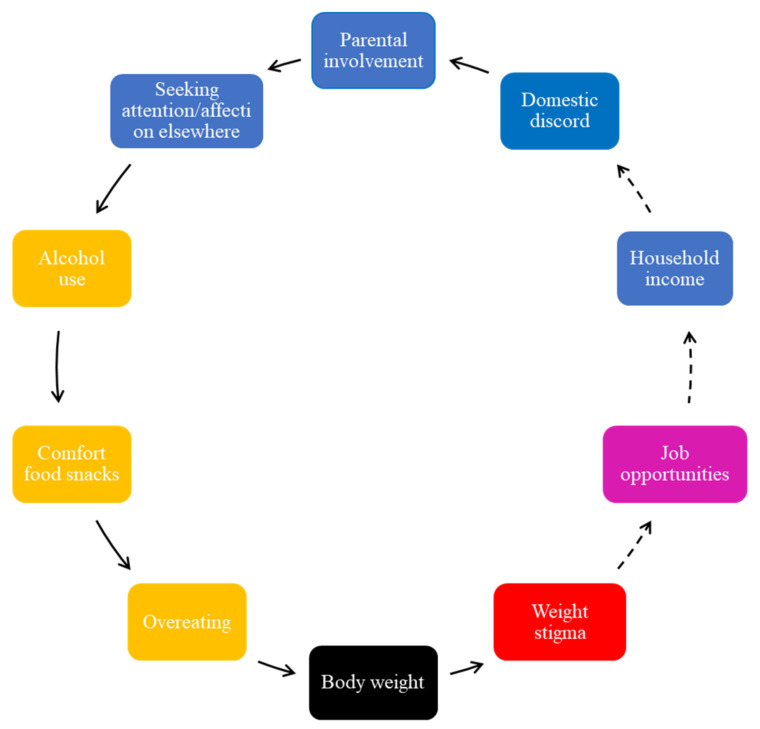
Parental involvement and unhealthy food intake.

Adolescents perceived the role of poor mental health in weight gain, including external stressors, such as parental neglect, to further perpetuate unhealthy diets; therefore, this is another FBL (Figure 6).

In Figure 6, unhealthy food intake was perpetuated by home environmental stressors, such as parental involvement. The adolescents reported that parents failing to respond to their emotional needs results in them seeking attention and affection elsewhere (Figure 6). Lack of parental involvement was viewed as insufficient, where time encouraging their children to exercise and monitoring their food intake or providing a healthy diet were the most commonly occurring themes in this respect. A lack of time and working long hours was cited as a reason why they thought their parents did not encourage healthy eating or prepare healthy home-cooked meals. It was indicated that they were exposed to irregular meal times, unhealthy snacking, and the purchasing of convenience foods. However, they mentioned that they did not blame their parents for working long hours as they were aware that work is a family responsibility and beyond their parents’ control. Generally, adolescents reported feeling concerned about weight stigma because it was experienced in negative ways by those overweight or obese and was associated with fewer job opportunities and lower income or wages. 

As indicated in Figure 1, the adolescents reported that attention-seeking behaviors, as a result of a lack of parental involvement, contribute to unplanned sexual relations, and places young people at risk of teenage pregnancies. While teenage pregnancies were described as highly prevalent in their communities, the adolescents also indicated that the use of contraceptives had an impact on their eating patterns, changed their appetite, and increased their body weight (Figure 1). 

They further reported that ineffective communication skills and lack of trust by parents further impacted their eating and drinking patterns (Figure 6). Adolescents mentioned that they preferred socializing with peers, consuming alcohol and unhealthy foods. This, they reported, would further increase their body weight.

The adolescents mentioned that parents’ working hours forced adolescents to become primary caregivers of younger siblings (Figure 1). They reported that low-income households consume lower overall nutritional quality food than high-income families and are further confronted with weight-related diseases, associated health-related morbidities, and additional psychosocial challenges.

## 4. Discussion 

This study constitutes a novel approach to the persistently problematic prevalence of adolescent obesity, particularly by exploring adolescents’ perception of the determinants of obesity in South Africa and illustrating the connections between factors that contribute to body weight changes. The results of this study illustrate that (i) physical activity and social media use; (ii) physical activity, health-related morbidity, and socio-economic status; (iii) accessibility of unhealthy food and energy intake/body weight; (iv) psychological distress, body weight, and weight-related bullying; and (v) parental involvement and unhealthy food intake are among the significant drivers influencing adolescent obesity.

First, adolescents’ excessive social media use may directly impose severe limitations on physical activity. Social media use was mentioned by all groups irrespective of SES. They mentioned that the body image ideals portrayed by social media are different to cultural body image expectations driving weight loss/gain and impacting their emotional wellbeing. Similarly to Savona et al. [14], in European countries, the role of factors, such as social media, particularly the influence of influencers, has been shown to be an important determinant of obesity [14]. In line with previous research [19,20], adolescents spend significant amounts of time using social media within the home and spend less time on outdoor activities. In addition, community safety appeared to be a driving factor in the adolescents’ choice of not participating in outdoor activities and their preference of staying indoors in South Africa, but these findings were not replicated in European countries. Thus, our study reinforces the need to establish whether social media can be an effective tool to encourage greater levels of physical exercise, and if so, how best to target safe environments, especially in this particular cohort. 

Second, easy accessibility to unhealthy foods in the local communities as a result of informal food outlets and large food manufacturers was consistently highlighted by the adolescents whom we worked with. In South Africa, there is a high density of food traders and a large quantity of such outlets in the vicinity of low-income communities [21], especially because of rapid urbanization-associated shifts [21], thus further perpetuating the problem of overweight and obesity. In European countries, adolescents indicated the influence of large food manufacturers and advertisers as important drivers of the consumption of unhealthy food [22]. Others have reported a high preponderance of fast-food establishments stimulating easy purchasing of unhealthy food [22,23,24], and thus consumption of high-calorie and low-nutrient food [25,26,27,28]. 

Third, it is worth considering in this study the impact of psychological distress on unhealthy food intake, body weight, and weight-related bullying. In European countries, mental health-related eating patterns, including compulsive, addictive, binge, or comfort eating, was often cited as a way of coping with stress [17]. Others have reported that psychological distress has contributed to a significant increase in calorie-dense junk foods and subtle food addiction to alleviate uncomfortable psychological and emotional states [29]. However, there are currently insufficient mental health interventions to assist adolescents, especially in South Africa [30], and the data emerging from this study augments the need for relevant interventions within the education sector.

Fourth, the perceived influence of parental involvement on young people’s food intake and risk of obesity highlights the family environment as being particularly significant for adolescents’ healthy behaviors. Contrary to the findings by Savona et al. [17], in this study, the adolescents were more likely to consume alcohol with peers as a way of seeking attention outside the home environment Previous work found that the lack of parental involvement and time available increases the risk of many physical and psychosocial challenges among adolescents, including addiction, suicide, teenage pregnancies, type 2 diabetes, and obesity [31]. Particularly in South Africa, many parents work nonstandard hours and are absent for a long period of time [32] while schools mostly close by 1 to 2 pm. Thus, potential programs need to acknowledge and accommodate parents’ long working hours and further explore interventions within a psychosocial framework. 

The findings of this study should be interpreted considering several limitations, including the fact that they cannot be generalized beyond the time, place, and participants of this study’s findings. While this study investigated adolescent perceptions, these questions were administered only to a subset of the population (i.e., 16–18-year-old adolescents within a school setting), and thus differences in their understanding of overweight and obesity may have influenced the types of drivers that they identified. 

## 5. Conclusions 

Despite these limitations, our study yields meaningful, complex, and policy-relevant insights into the drivers of obesity in a South African context. This approach, both the process of construction and the final visualization of the CLD, provides a basis for developing prevention and intervention recommendations for adolescent overweight and obesity. 

## Figures and Tables

**Figure A1:**
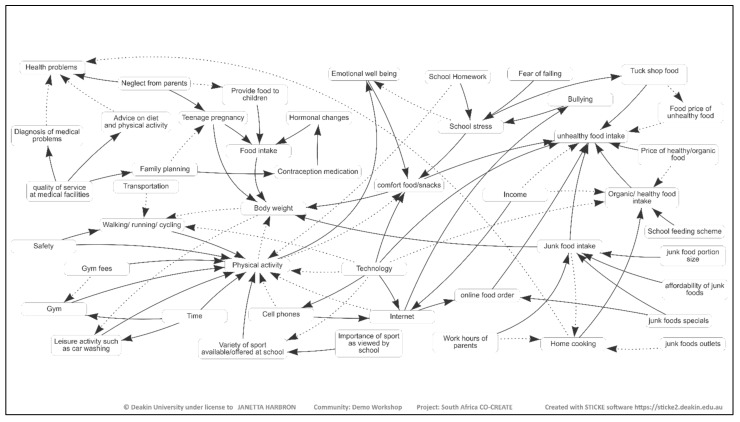
School map 1#.

**Figure A2:**
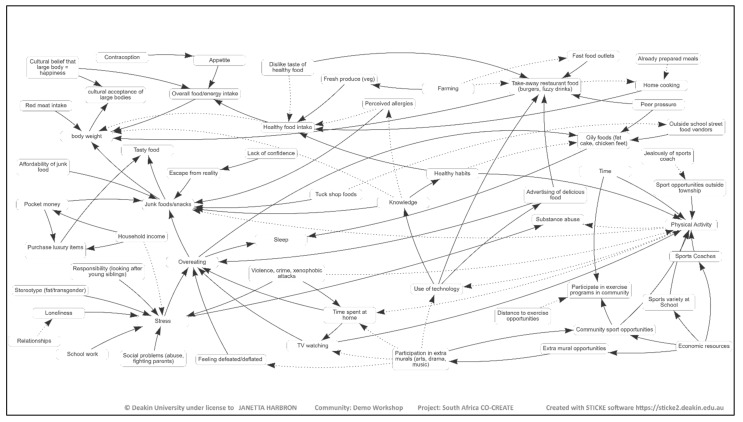
School map 2#.

**Figure A3:**
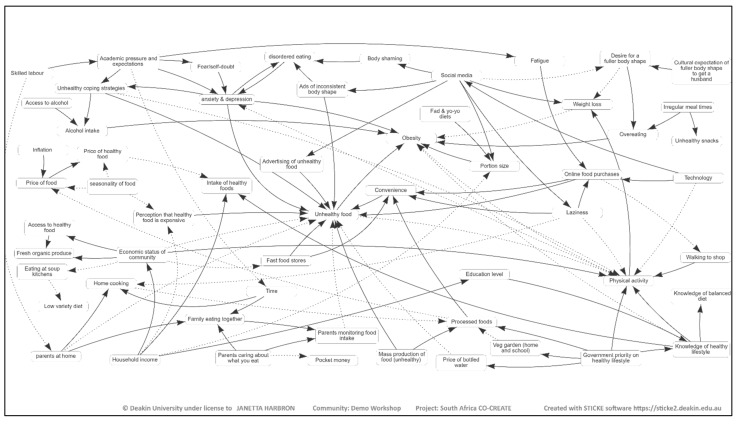
School map 3#.

**Figure A4:**
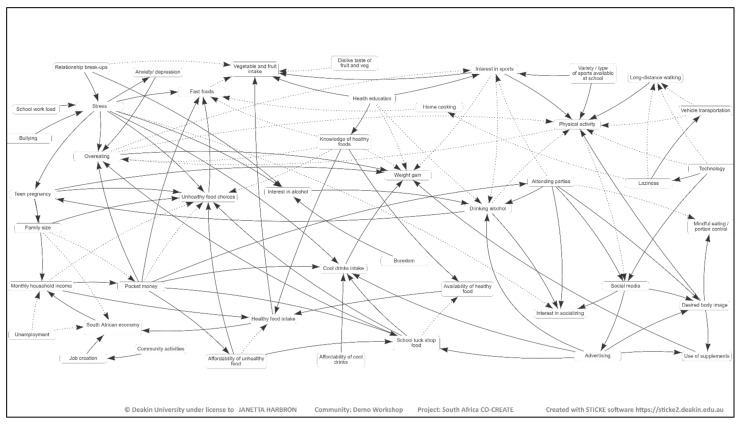
School map 4#.

## Data Availability

Data is contained within the article.

## Appendix A

### Feedback Loop Descriptions

1. Physical activity → motivation to exercise → staying indoors → outdoor sports → physical activity
2. Physical activity → motivation to exercise → staying indoors → use of technology à physical activity
3. Use of technology → knowledge about healthy lifestyle → impact of knowledge on healthy lifestyle → awareness of the benefits of healthy lifestyle → easiness to share information on healthy lifestyle → use of technology
4. Use of technology → social media use → exposure to food advertising → advertising of unhealthy food → impact of knowledge on healthy lifestyle → awareness of the benefits of healthy lifestyle → easiness to share information on healthy lifestyle → use of technology
5. Use of technology → physical activity → motivation to exercise → staying indoors → use of technology
6. Stress → anxiety/depression → disordered eating/emotional eating → comfort food/snacks → overeating → unhealthy food intake → body weight → weight-related bullying → pressure on particular body image → self-esteem → stress
7. Stress → anxiety/depression → disordered eating/emotional eating → comfort food/snacks → overeating → body weight → weight-related bullying → pressure on particular body image → self-esteem → stress
8. Stress → alcohol use → comfort food/snacks → overeating → unhealthy food intake → body weight → weight-related bullying → pressure on particular body image → self-esteem → stress
9. Stress → alcohol use → comfort food/snacks → overeating → body weight → weight-related bullying → pressure on particular body image → self-esteem → stress
10. Stress → alcohol use → comfort food/snacks → overeating → body weight → weight-related bullying → pressure on a particular body image → self-esteem → stress
11. Alcohol use → comfort food/snacks → overeating → body weight → weight stigma → job opportunities → household income → domestic discord → parental involvement → time pressure → stress → alcohol use
12. Household income → neighborhood socioeconomic status → accessibility of unhealthy food → purchasing of unhealthy food intake → unhealthy food intake → body weight → weight-related diseases → health-related morbidity → household income
13. Household income → domestic discord → parental involvement → seeking attention/comfort elsewhere → alcohol use → comfort food → overeating → body weight à weight stigma → job opportunities → à household income
14. Teenage pregnancy → body weight → weight stigma → job opportunities → household income → domestic discord → parental involvement → seeking attention/affection elsewhere → teenage pregnancy
